# Scalable Fabrication of Highly Organized, Horizontally Aligned Sub‐5 nm Silicon Nanowires via Chemical Vapor Etching

**DOI:** 10.1002/smsc.202400627

**Published:** 2025-02-28

**Authors:** Juyeon Seo, Peiyun Feng, Jianlin Li, Sanghyun Hong, Sen Gao, Ji Young Byun, Yung Joon Jung

**Affiliations:** ^1^ Department of Mechanical and Industrial Engineering Northeastern University Boston Massachusetts 02115 USA; ^2^ Extreme Materials Research Center Korea Institute of Science and Technology 5 Hwarang‐ro 14‐gil Seoul 02792 Republic of Korea

**Keywords:** chemical vapor etchings, hierarchical structures, silicon nanowires, surface‐enhanced raman scatterings

## Abstract

Herein, the scalable fabrication of hierarchical silicon structures featuring high‐density, horizontally super‐aligned sub‐5 nm silicon nanowires (SiNWs), is reported. These unprecedented, highly organized silicon architectures with tunable sizes and densities are fabricated using straightforward micro‐patterned SiO_2_/Si templates followed by a chemical vaporetching process. In time‐resolved structural analysis, it is revealed that rapid, aggressive etching is crucial for creating an inhomogeneous spatial distribution of vapor etchants, inducing surface defects acting as preferential sites for localized anisotropic silicon etching along <111> direction. The efficacy of this unique structure is demonstrated as a single‐molecule detectable surface‐enhanced Raman scattering sensor, incorporating sub‐10 nm silver plasmonic nanoparticles. Its distinct structural features—marked by quantum‐confined dimensions, ultrahigh surface area, dual‐scale roughness, and exceptional uniformity—enable significant enhancement of optical response and detection sensitivity down to 10^−11^ m. These highly controlled sub‐5 nm SiNW architecture can broaden the applications of quantum nanowires in chemical and bio‐sensing and other emerging technologies.

## Graphical abstract

An unprecedented silicon nano/micro‐hierarchical architecture featuring sub‐5 nm quantum silicon nanowires (SiNWs) is achieved via a single‐step, catalyst‐free chemical vapor etching process. Using a simple micro‐patterning technique, a scalable strategy for fabricating highly ordered SiNWs/micro‐pit arrays is developed. In the results, their exceptional potential is highlighted as an efficient template for single‐molecule detection.

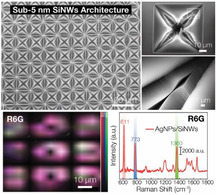

## 1 Introduction

The synthesis of sub‐5 nm silicon nanowires (SiNWs) and their integration into highly organized 2D and 3D architectures represent significant challenges in the field of nanotechnology. As devices continue to shrink in size, the ability to fabricate SiNWs at these ultrasmall dimensions is critical for unlocking new physical properties driven by quantum confinement effects. At diameters below 5 nm, SiNWs exhibit tunable electronic, optical, and mechanical characteristics, making them promising candidates for next‐generation applications in nanoelectronics, quantum computing, energy storage, and optoelectronics.^[^ ^1^, ^2^, ^3^, ^4^, ^5^, ^6^ ^]^ However, achieving precise control over nanowire dimensions and their arrangement into desired structures remains an ongoing challenge, both in bottom‐up and top‐down fabrication techniques.

Traditional catalyst‐assisted methods, including vapor–liquid–solid (VLS) growth and metal‐assisted chemical etching (MACE), can produce SiNWs with controlled diameters and alignment.^[^ ^7^, ^8^, ^9^, ^10^, ^11^, ^12^, ^13^, ^14^ ^]^ However, SiNWs synthesized by these methods typically have large diameters (10–100 nm), where quantum confinement effects are diminished. Valuable progress has been made in synthesizing smaller SiNWs through various approaches, such as supercritical solution‐phase growth with Au nanoclusters,^[^ ^1^ ^]^ etching oxide sheaths from core–shell SiNWs,^[^ ^15^ ^]^ and VLS growth and MACE processes using small catalyst metal nanoparticles.^[^ ^16^, ^17^, ^18^, ^19^, ^20^, ^21^ ^]^ However, these approaches rely on catalyst nanoparticles, necessitating additional post‐processing steps for their removal. Additionally, the synthesized nanowires are typically in vertical or random configurations with lower density, which require complex post‐growth assembly techniques such as direct transfer,^[^ ^22^, ^23^, ^24^ ^]^ dielectrophoretic,^[^ ^25^, ^26^ ^]^ and fluidic assembly (Langmuir–Blodgett)^[^ ^27^ ^]^ methods to build SiNW‐based network architectures for broader applications. In contrast, conventional lithography‐based top‐down approaches can precisely control the dimensions, locations, and arrangements of nanowires through lithographic patterning and dry/wet etching of bulk silicon (Si).^[^ ^28^, ^29^, ^30^, ^31^, ^32^ ^]^ However, the high‐density fabrication of sub‐5 nm SiNWs and the creation of their organized 2D–3D architectures remain challenging due to lithography's inherent resolution limits and complexity. Recently, 3D multilevel lateral and vertical configurations of SiNW‐based architectures have been demonstrated by combining conventional lithography and etching processes followed by catalyst deposition and subsequent growth of SiNWs.^[^ ^33^, ^34^, ^35^, ^36^ ^]^ Using traditional lithography allows the structural design of the groove template where the nanowires with controlled sizes, pitches, and densities can be grown and integrated on a large scale. However, multiple preparation steps are needed prior to growth, and further optimized process development is required to achieve the sub‐5 nm nanowire diameter.

Here, we present a unique approach to synthesizing highly organized, horizontally aligned sub‐5 nm SiNW‐based hierarchical architectures by controlling silicon‐etching kinetics in a chemical vapor etching (CVE) process. This CVE method builds on our previous research, where silicon tetrachloride (SiCl_4_) and hydrogen chloride (HCl) vapors were utilized as primary vapor etchants to transform Si wafers into highly dense and vertically aligned SiNWs without the need for metal nanoparticle catalysts.^[^ ^37^, ^38^ ^]^ Our findings demonstrate the formation of high‐density and horizontally aligned sub‐5 nm SiNW arrays, self‐organized within microscale‐inverted pyramid pits through a single‐step vapor etching process. Time‐resolved structural analysis reveals that rapid and vigorous etching conditions are essential for generating a nonuniform spatial distribution of vapor etchants, which induces initial defects that act as nucleation sites for continuous anisotropic etching along the <111> direction, leading to the formation of densely packed horizontal nanowire arrays. Building on the formation mechanism uncovered in this study, we have developed a generic strategy to fabricate highly ordered and well‐defined arrays of horizontal SiNWs/micro‐pit structures. These Si hierarchical structures’ location, size, and density were precisely engineered by simple micro‐patterning silicon dioxide (SiO_2_) on Si wafer surfaces. To demonstrate the practical application, we have integrated plasmonic silver (Ag) nanoparticles (AgNPs) into the sub‐5 nm Si nano‐micro‐hybrid architecture, achieving single‐molecule detection sensitivity in surface‐enhanced Raman scattering (SERS) and highlighting its potential as the functional platform for advanced plasmonic devices.

## 2 Results and Discussion

### 2.1 Formation of the Si Nano/Micro‐Hierarchical Structure

**Figure 1:**
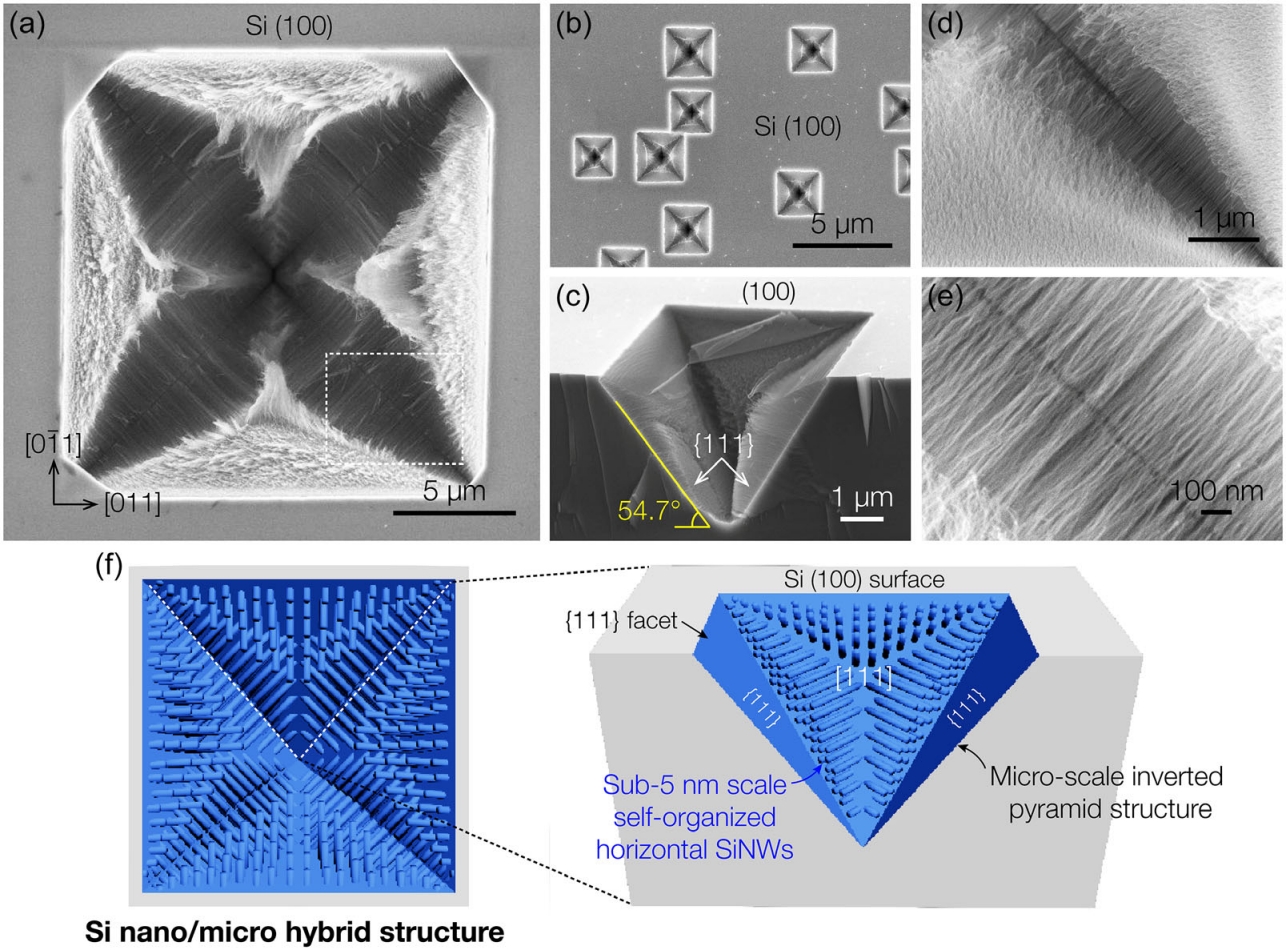
Morphological characteristics of the sub‐5 nm SiNW‐based hierarchical structures. a) Top‐view SEM image of the Si nano/micro‐hierarchical structure, highlighting the self‐organized SiNWs within the microscale Si etch pit. The pit edges are oriented along high‐symmetry <110> directions. b) Low‐magnification SEM image showing the random distribution of microscale Si etch pits across the Si (100) surface. c) Tilted SEM image of the SiNWs/inverted pyramid pit structure, presenting densely packed nanowire arrays forming a tetrahedral configuration on each {111} facet. d,e) Enlarged SEM images of the region outlined by the white‐dashed line in (a), revealing horizontally aligned nanowires within the etch pits. f) Schematic illustration of the Si nano/micro‐hybrid architecture, showing high‐density SiNW arrays arranged in three orientations on each Si {111} plane of square‐shaped micro‐pits.

**Figure** **1**a shows a scanning electron microscopy (SEM) image of a self‐organized ultra‐narrow SiNW‐based hierarchical structure formed on a Si (100) wafer surface by a single‐step vapor phase etching of silicon. The vapor phase silicon etching process, developed in our previous works, leverages the anisotropic etching of the bulk Si wafer into vertical nanowire structures using the HCl and SiCl_4_ vapors as primary etchants without the assistance of metal nanoparticles.^[^ ^37^, ^38^ ^]^ In this study, we have established rapid and vigorous etching conditions by increasing the carrier gas flow rate and significantly shortening the reaction time. These conditions promote the uneven localization of vapor etchants in regions where the native oxide is more thermally decomposed and weakened, targeting specific attack sites on the silicon surface and facilitating the formation of this unique silicon nano/micro‐hierarchical structure. Figure 1b shows square‐shaped micro‐pits, randomly distributed on the Si (100) surface along high‐symmetry <110> directions, which contain densely packed ultra‐narrow nanowire arrays. Unlike the vertical nanowires, which form uniformly on the Si surface (Figure S1, Supporting Information), we observed that the highly dense SiNWs align horizontally within inverted pyramid‐shaped micro‐pits (Figure 1c). The pit sidewalls are inclined at an angle of 54.7° with respect to the (100) surface, indicating that the square pits are bounded by Si {111} facets. This structural characteristic arises from localized anisotropic etching by HCl and SiCl_4_ vapors, which will be discussed in more detail later. Figure 1d–f depicts the orientation and arrangement of the horizontally aligned nanowires within the micro‐pits. The CVE preferentially occurs perpendicular to the exposed {111} surfaces, producing SiNW arrays aligned in the <111> direction. As the reaction progresses, the <111> nanowires, formed on each {111} plane, align along three distinct directions from the centroid of the {111} triangular facets due to van der Waals (vdW) interactions, given from aligned SiNWs with ultrathin diameters of less than 5 nm. Further details are discussed in Figure 3.

### 2.2 Formation Mechanism of the Si Nano/Micro‐Hierarchical Structure

**Figure 2:**
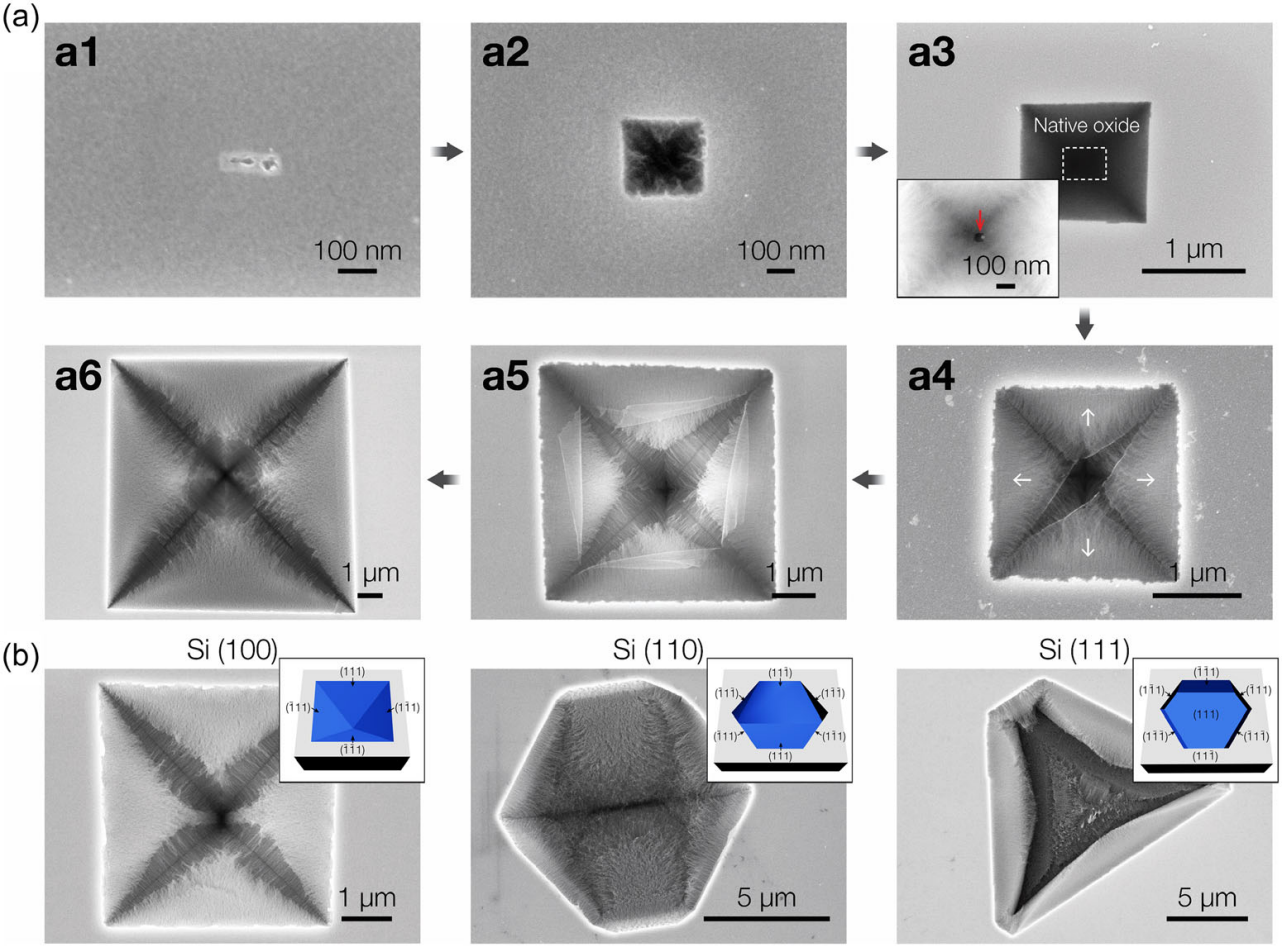
Time‐resolved morphological evolution of the SiNW/micro‐pit structures. a) Time‐dependent morphological evolution of the horizontally aligned SiNWs within an inverted pyramid structure on the Si (100) surface. The process is initiated by defective sites caused by rapid and aggressive etching conditions. Vapor etchants penetrate through cracks in the native silicon oxide layer (marked by the red arrow in Figure 2a3), continuously etching the exposed Si {111} facets to form nanowire structures. White arrows in Figure 2a4 show the delamination of the native silicon oxide layer over time. As vapor etchants are supplied, the nanowires elongate, resulting in the progressive enlargement of microscale etch pits. b) Top‐view SEM images of SiNWs organized inside etch pits on the Si {100}, {110}, and {111} surfaces after removing the native oxide layer with adhesive tape. The insets are schematic illustrations of representative etch pit structures for the Si with different crystallographic orientations, showing that the pits are terminated with {111} facets.

To elucidate the formation mechanism of this unique Si hierarchical structure, we investigated its morphological evolution by varying the CVE reaction time from 0 to 3 min while keeping the other parameters constant (**Figure** **2**a). At the initial stage, small, shallow cavities begin to form on the Si (100) surface (Figure 2a1). These newly generated defective sites, which are energetically favorable for etching, serve as active locations for the vapor phase etchants, eventually evolving into square‐shaped pits (Figure 2a2). The kinetics of silicon etching are governed by the removal rate of Si atoms, which is heavily influenced by the bond structure of different crystallographic planes.^[^ ^39^, ^40^ ^]^ On the {100} plane, each Si atom is bonded to two internal atoms through Si—Si back bonds and possesses two dangling bonds. In contrast, atoms on the {111} plane are connected by three back bonds and have only one dangling bond. Importantly, the higher atomic density and three equivalent back bonds make the {111} planes the most stable among the crystallographic orientations. As detailed in Table S1, Supporting Information, the surface bond densities for the Si {100}, {110}, and {111} planes are ≈1.36, 0.96, and 0.78 × 10^15^ cm^−2^, respectively, corresponding to surface free energies of 1.99, 1.41, and 1.15 J cm^−2^.^[^ ^41^, ^42^ ^]^ This indicates that surface atoms on the {100} planes are more reactive to the HCl and SiCl_4_ etchants, leading to a faster etching rate along the <100> direction. Consequently, the etching process continues until the more stable {111} planes with lower surface energy are exposed. This anisotropic behavior leads to the formation of characteristic square‐shaped, inverted pyramidal structures bounded by {111} facets with either pointed or truncated edges (Figure S2, Supporting Information). Once these small pits are formed during the initial stage, vapor etchants penetrate through cracks in the native silicon oxide (SiO_*X*_) layer (inset of Figure 2a3), initiating the etching of {111} facets into nanowire structures and gradually enlarging the inverted pyramid pits over time. The amorphous native oxide layer, being more resistant to the etchants than silicon, acts as a mask, confining the vapor within the pits and protecting the newly formed nanowires from being etched away (Figure S3, Supporting Information). As the reaction progresses, however, the vapor etchants begin to remove the oxide layer (Figure 2a4–a6). This process gradually increases the number of pits filled with ultrahigh‐density nanowire arrays, eventually covering almost the entire substrate surface. As adjacent etch pits merge, the structural collapse accelerates anisotropic etching along the [100] direction, leaving behind vertical nanowire arrays (Figure S4, Supporting Information).

We conducted the same CVE reactions on Si wafers with different crystallographic orientations to validate the proposed etching mechanism. Figure 2b displays SEM images of the resulting SiNW/micro‐pit structures on <100>, <110>, and <111>‐oriented Si surfaces after removing the residual native amorphous oxide layer using adhesive tape. Inverted pyramidal, hexagonal, and triangular pit shapes were observed for the {100}, {110}, and {111} planes, respectively, consistent with the expected bare pit structures terminated by {111} planes due to the slower etching rate along the <111> direction (insets of Figure 2b).^[^ ^43^, ^44^ ^]^ Furthermore, it was confirmed that vapor etchants penetrated the cracked SiO_*X*_ masking layer, subsequently etching the exposed {111} facets into nanowire structures for all orientations (Figure S5, Supporting Information). These observations suggest that the etch pit nucleation, driven by rapid and aggressive etching conditions, is crucial for forming horizontally aligned nanowire‐based hierarchical structures.

### 2.3 Crystalline Structure of the Horizontally Aligned Sub‐5 nm SiNWs

**Figure 3:**
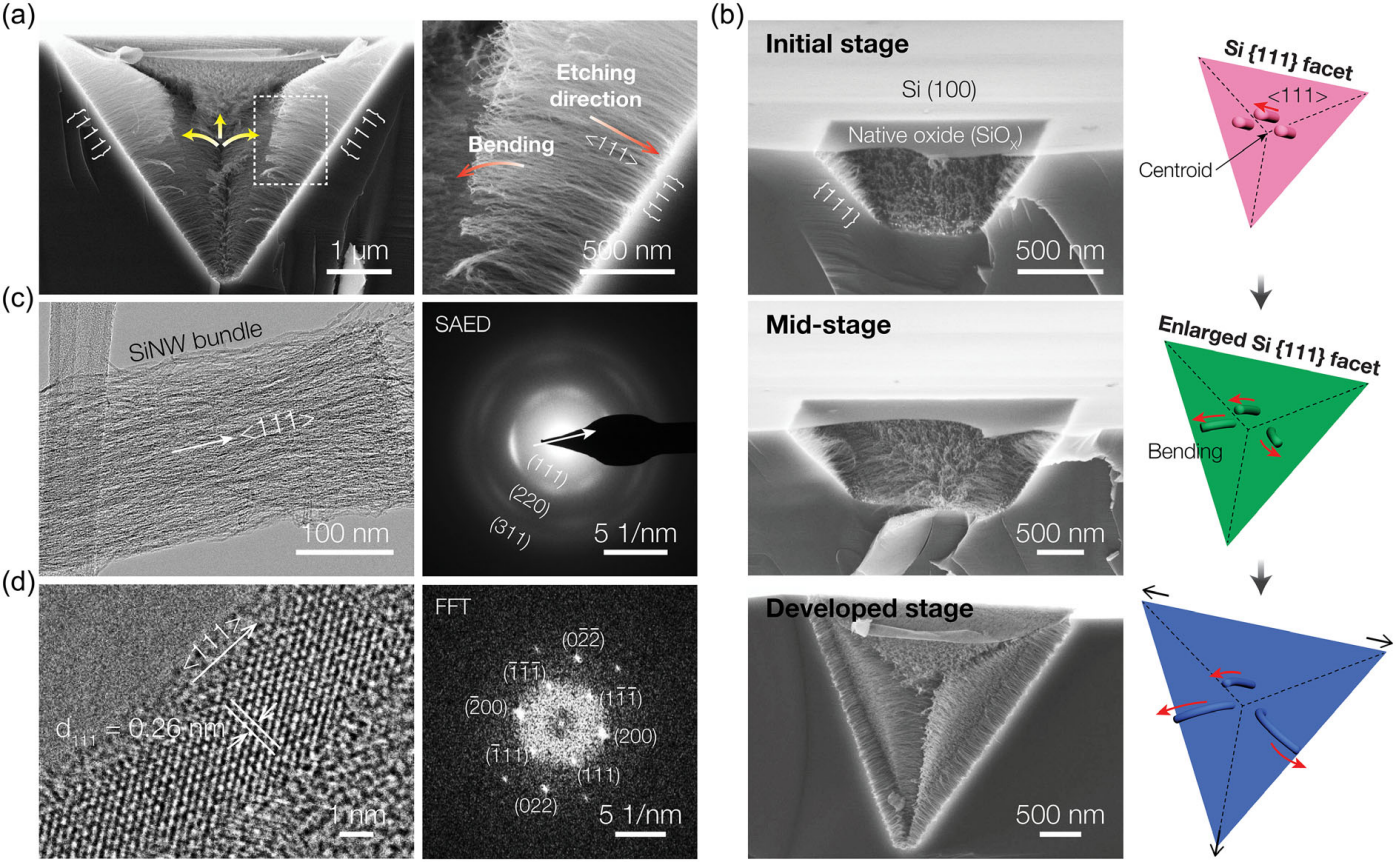
Crystalline structure of the horizontally aligned sub‐5 nm SiNWs. a) Cross‐sectional SEM images of the SiNWs/micro‐pit structure. Yellow arrows in the low‐magnification image highlight the nanowire arrangement on each Si {111} plane. The magnified image suggests that the etching predominantly occurs perpendicular to the Si {111} surfaces. b) Tilted‐view SEM images of severed micro‐pits (left) and corresponding schematic illustrations of the evolution of the nanowire arrangement on each Si {111} plane (left) as the pits enlarge overtime. c) Low‐magnification HRTEM image (left) and corresponding SAED pattern (right) of the aligned SiNW bundle detached from the pits. The white arrows indicate that the overall crystalline orientation of the as‐formed nanowires is <111>. d) High‐magnification HRTEM (left) and corresponding FFT (right) images of the individual SiNW showing its high crystallinity with a diameter in the sub‐5 nm scale. All HRTEM images and SAED pattern were taken along the [110] zone axis.

**Figure** **3**a shows cross‐sectional SEM images of the silicon nano/microstructure, highlighting the etching of triangular {111} facets into nanowire arrays. These nanowires extend along three distinct directions from the centroid (marked with yellow arrows in Figure 3a) due to vdW forces generated by the high‐density nanowire alignment to reduce the total surface energy.^[^ ^45^ ^]^ Despite these multidirectional extensions, the nanowires maintain a predominant alignment along the <111> direction, perpendicular to the {111} plane. A closer view reveals that the nanowires remain vertical at the root, while their tips deviate noticeably from the <111> orientation, exhibiting curvature (Figure 3a, right). The pit evolution over time, shown in Figure 3b, further demonstrates that as the {111} surfaces are etched deeper toward <111> directions, accompanied by an increase in nanowire density, the nanowires—initially stand normal to {111} planes—tend to deflect and eventually align along three distinct directions radiating from the centroid of the triangular surfaces (Figure S6, Supporting Information). Additionally, we observe a depth‐dependent variation in nanowire length, with longer nanowire arrays concentrated in the central region of the triangular {111} surface (Figure 3a). In contrast, there is a gradual decrease in length toward both the apex and base regions of the inverted pyramid etch pit, corresponding to its depth profile of <111> direction. The crystalline structure of the nanowires, formed on {111} sidewalls, was characterized using high‐resolution transmission electron microscopy (HRTEM). Selected area electron diffraction (SAED) patterns of the horizontally aligned SiNW bundles, obtained along the [110] zone axis, confirm the high crystallinity of the nanowires, which preferentially form along the <111> direction (Figure 3c and S7, Supporting Information). The three distinct diffraction rings, corresponding to ≈2.51, 1.53, and 1.32 Å, are indexed as the {111}, {220}, and {311} planes of the diamond‐cubic Si lattice with the space group Fd3m, respectively.^[^ ^46^ ^]^ The estimated lattice parameter of the nanowires is 4.32 Å, which represents about 79.6% of that of bulk silicon (*a* = 5.43 Å). This finding, alongside our previous results on <100 > SiNWs, further corroborates the significant lattice contraction (≈20%) observed in the CVE‐formed nanowires, attributed to the unique etching chemistry of the CVE process and their ultrasmall dimensions, regardless of crystalline orientation.^[^ ^37^, ^38^ ^]^ The magnified HRTEM image in Figure 3d further illustrates that the nanowires possess a sub‐5 nm diameter, with clear lattice fringes exhibiting an interlayer spacing of 0.26 nm, corresponding to the Si (111) plane. The corresponding fast Fourier transform (FFT) image also clearly indicates that the axial orientation of the nanowires aligns with the <111> direction, consistent with the orientation of the etched {111} surfaces.

### 2.4 Controlled Fabrication of the Si Nano/Micro‐Hierarchical Structure

**Figure 4:**
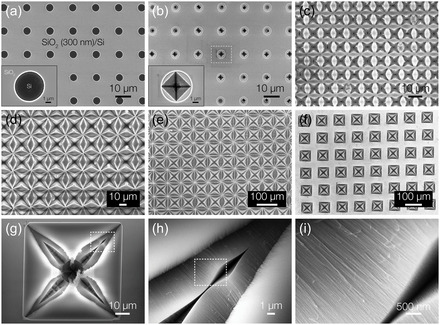
Controlled fabrication of the Si hybrid structure using a facile template‐based CVE process. a,b) Low‐magnification SEM images of (a) Si micro‐hole arrays fabricated on the SiO_2_ (300 nm)/Si wafer and b) SiNWs/micro‐pit arrays locally formed at exposed Si hole patterns in the early stage of the CVE process. The insets are the enlarged images presenting the 5 μm hole before and after the CVE reaction. The inset image in (b) shows SiNWs/micro‐pits covered by a cracked native oxide layer, supporting the proposed etching mechanism. c–f) Top‐view SEM images of self‐organized SiNW‐based nano/micro‐hierarchical structure with varying sizes, densities, and spacings, prepared by using micro‐patterned templates with varying hole spacings: (c) 10 μm, (d) 20 μm, (e) 60 μm, and (f) 100 μm. The Si hole size was kept constant at 5 μm for all conditions. g–i) Enlarged images of individual micro‐pits containing densely packed, super‐aligned sub‐5 nm horizontal SiNW arrays, demonstrating the successful evolution of Si micro‐holes into highly ordered and uniform Si nano/micro‐hybrid architectures.

Our observations suggest that etch pit nucleation is driven by localized anisotropic etching, forming well‐organized, highly dense, and horizontally aligned SiNW arrays with sub‐5 nm dimensions within micro‐pits. Based on this finding, we have developed a scalable and controllable method to fabricate this sophisticated nano/micro‐architecture. The 5 μm Si hole arrays patterned on the SiO_2_ (300 nm)/Si wafer were first prepared as the CVE reaction template via a simple lithography‐based micro‐patterning and dry etching of SiO_2_ (see Experimental Section for details) (**Figure** **4**a). We hypothesized that the surface characteristics of silicon after SiO_2_ dry etching would enable each bare Si window to act as a selective site for surface defect creation, ultimately evolving into a SiNWs/micro‐pit hybrid structure. Specifically, we assumed that the growth of native oxide would be slightly delayed after the dry etching process,^[^ ^47^, ^48^ ^]^ rendering each Si hole more susceptible to vapor etchants compared to the surrounding SiO_2_ mask, thus facilitating defect formation. Additionally, structural damage induced by energetic ions during dry etching was expected to further accelerate pit nucleation.^[^ ^49^, ^50^ ^]^ Figure 4b shows the as‐patterned SiO_2_/Si template following a 5 min CVE reaction, revealing the formation of square‐shaped etch pits, covered by cracked native oxide, at each exposed Si (100) micro‐window (the enlarged image is shown in Figure S8, Supporting Information). This result confirms our hypothesis regarding the Si hole patterns acting as active centers for localized anisotropic etching. Crucially, the simplicity of the template fabrication process allows for precise control over the location, size, and density of the SiNWs/micro‐pit arrays, enabling the design of customized architectures for specific applications. As shown in Figure 4c–f and S9, Supporting Information, by adjusting the inter‐hole spacing from 10 to 100 μm and optimizing the reaction time accordingly, we achieved highly ordered and uniform Si nano/microstructure arrays with controlled size, density, and spacing. The magnified images of individual micro‐pits in Figure 4g–i reveal remarkable structural features, showcasing densely packed and super‐aligned sub‐5 nm nanowire arrays neatly organized within the square‐shaped etch pits.

### 2.5 SERS Sensing Capability of the Si Nano/Micro‐Hierarchical Structure

**Figure 5:**
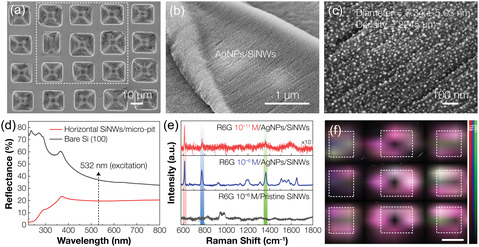
SERS detection capability of AgNP‐decorated SiNWs/micro‐pit structure. a) Top‐view SEM image of AgNP‐coated SiNWs/micro‐pit arrays prepared as the SERS template for R6G detection. b,c) Magnified SEM images of AgNP‐decorated SiNW arrays organized within micro‐pits. The sub‐10 nm spherical AgNPs are densely distributed across the nanowire arrays. d) Reflectance spectra of the bare Si (100) wafer (gray) and horizontal SiNWs/micro‐pit arrays (red). The hybrid structure exhibits improved light‐trapping performance due to its multiscale surface features. e) Representative SERS spectra of the pristine SiNWs with 10^−6^ m R6G (gray) and AgNP‐decorated SiNWs/micro‐pit arrays with 10^−6^ m (blue) and 10^−11^ m R6G (red). Highly amplified Raman signals can be achieved by incorporating ultrahigh‐density AgNPs. f) Raman map of R6G (10^−6^ m) by targeting the Raman shift at 611 cm^−1^ (red), 773 cm^−1^ (blue), and 1360 cm^−1^ (green), acquired from the white‐dashed area in (a), representing the strong and uniform Raman signal coming out from each Si hierarchical structure. The white‐dashed line area indicates the individual etch pit. The scale bar is 10 μm.

The construction of nano/micro‐hierarchical structures has been shown to enhance the optical response to incident electromagnetic waves, making them highly effective for various plasmonic device applications.^[^ ^51^, ^52^, ^53^, ^54^ ^]^ In this work, we demonstrate the efficacy of our unique Si nano/micro‐hierarchical architecture as a template for plasmonic enhancement by fabricating a SERS sensor. AgNPs were incorporated into the hierarchical structure to induce localized surface plasmon resonance, significantly amplifying the local electromagnetic field. **Figure** **5**a–c displays the overall morphology of the SiNW arrays decorated with AgNPs (see Experimental Section for details). Small, spherical AgNPs, with an average diameter of 7.34 ± 5.03 nm and an areal density of 2245 μm^−2^, were uniformly distributed across the nanowires (see Note S1, Supporting Information, for measurement details). This ultrahigh‐density distribution and small interparticle spacing suggest that the structural characteristics of the SiNWs—namely, their surface roughness, ultrathin nature, and high aspect ratio (exceeding 10,000)—make them ideal for supporting AgNP nucleation (Figure S10, Supporting Information). These features promote nanoparticle formation across their entire surface while limiting the growth of the AgNPs to dimensions on par with the nanowires themselves (Figure S11, Supporting Information). The result is an abundance of interparticle hotspots attributed to the densely packed plasmonic AgNPs, which are expected to intensify the Raman signals significantly. Reflectance spectra in Figure 5d reveal that the nano/microstructured surface improves light‐trapping performance compared to a bare Si wafer across the visible spectrum. This result can be attributed to the multiscale structural features of the SiNW/micro‐pit structures. Specifically, the incident light undergoes multiple scattering and reflections within the angled facets of the inverted pyramid pits, while the densely packed nanowire arrays, due to their subwavelength dimensions and interspacing, facilitate the efficient coupling of light into the nanostructured surface.^[^ ^55^, ^56^, ^57^ ^]^ These factors collectively enhance light confinement and strengthen the local electromagnetic field. Additionally, a marked decrease in reflectance in the UV region is observed for the SiNWs/micro‐pit arrays, likely attributed to the ultrawide bandgap properties of the sub‐5 nm SiNW arrays.^[^ ^37^ ^]^

To demonstrate the sensing capabilities of the AgNP‐coated Si hybrid structure, rhodamine 6G (R6G) was used as the probe molecule. Figure 5e compares the SERS signals obtained from pristine and AgNP‐decorated SiNWs/micro‐pit arrays at an excitation wavelength of *λ* = 532 nm. At 10^−6^ m, all characteristic Raman peaks of R6G molecules are clearly observable only from the AgNP‐decorated substrate,^[^ ^58^ ^]^ indicating that AgNPs are primarily responsible for the Raman signal enhancement. Remarkably, three prominent peaks at 611, 773, and 1360 cm^−1^ are detectable even at an extremely low concentration of 10^−11^ m. It should be noted that at low R6G concentrations, the polarization characteristics of the single molecule selectively enhance specific vibrational modes aligned with the polarization direction of the incident laser, thereby suppressing some fingerprint peaks of R6G.^[^ ^59^ ^]^ Given that the number of probe molecules within the effective Raman‐active area is estimated to be ≈1.35 for 10^−11^ m R6G under our experimental conditions (see Note S2, Supporting Information, for calculation details), this result suggests that the Si nano/micro‐hierarchical architecture‐based SERS substrate is capable of single‐molecule detection. The enhancement factor of the substrate 5.677 × 10^10^ (refer to Note S3, Supporting Information, for further details), which is comparable with the range of 10^8^–10^11^ observed in single‐molecule SERS detection.^[^ ^59^, ^60^, ^61^, ^62^ ^]^ To assess SERS signal uniformity, point‐by‐point Raman mapping was performed by measuring 143 points over 65 × 57 μm area of the AgNP‐decorated SiNWs/micro‐pit arrays. Figure 5f shows the Raman map of 10^−6^ m R6G, highlighting peaks at 611, 773, and 1360 cm^−1^, which correspond to the C—C—C in‐plane vibration, out‐of‐plane vibration of C—H bonds, and aromatic C—C stretching mode, respectively.^[^ ^63^, ^64^ ^]^ This result confirms that the micro‐pits with densely packed AgNP‐decorated SiNW arrays generate a strong and uniform Raman signal. These findings highlight the advantages of this nano/micro‐hierarchical structure as the template for SERS detection, attributable to several factors: first, the nano–microstructured surface effectively traps incident light and enhances the electric field within the valleys, as evidenced by a 17.3% reduction in reflectance at *λ* = 532 nm (Figure 5d). Additionally, the negligible visible light absorption by sub‐5 nm SiNWs, due to their ultrawide bandgap from quantum confinement effects,^[^ ^37^ ^]^ likely facilitates stronger light interactions between AgNPs. Second, the high density of interparticle hotspots generated by the nanowire structure contributes to substantial near‐field enhancement, significantly boosting SERS signal intensity. Third, the multiscale roughness of this hierarchically structured surface further amplifies the electromagnetic field, enhancing the SERS signal. Finally, the 3D geometry of the horizontally aligned SiNW arrays offers abundant binding sites for the analyte molecules, improving detection sensitivity. Beyond its ultimate single‐molecule sensitivity, the nano/micro‐hierarchical structure makes our SERS sensor stand out from others with a similar limit of detection.^[^ ^65^, ^66^, ^67^ ^]^ The highly ordered yet well‐separated SiNWs/micro‐pit arrays are expected to enable the site‐specific and selective detection of multiple analytes on a single substrate, enhancing their versatility for complex analytical applications in the biomedical field.^[^ ^68^, ^69^ ^]^

## 3 Conclusion

We have successfully developed the highly ordered Si nano/micro‐hierarchical architecture by controlling the reaction kinetics of the CVE process. The highly dense and horizontally aligned SiNW arrays with a sub‐5 nm diameter are self‐organized within the microscale‐inverted pyramid pits. Our time‐dependent morphological analysis reveals that the initial defect sites—resulting from the inhomogeneous spatial distribution of vapor etchants—serve as reactive sites that promote continued anisotropic etching along the <111> direction, leading to the formation of the horizontally aligned SiNWs/micro‐pit binary structure. Moreover, we have demonstrated that utilizing a simple micro‐patterned SiO_2_/Si template, followed by CVE process, enables the precise construction of uniform SiNWs/micro‐pit arrays with adjustable pit sizes and densities at predetermined locations. This innovative silicon nano/micro‐hierarchical architecture functions effectively as a scaffold for enhancing optical responses and detecting single molecules. The advantages of this architecture include an exceptionally large surface area, multiscale surface roughness, high periodicity, and a 3D spatial structure. We anticipate that this highly controlled SiNW‐based hierarchical architecture presents a promising material system for applications in chemical and bio‐sensing, energy systems, and other novel technologies.

## 4 Experimental Section

### Materials Preparation

The self‐organized SiNWs/micro‐pit hierarchical structure was fabricated using the CVE process.^[^ ^37^, ^38^ ^]^ The polished silicon wafer ((100)‐orientation, p‐type, resistivity 1–10 Ω cm; UniversityWafer, Inc.) was placed at the center of the quartz tube chamber, and a chemically resistant Viton fluoroelastomer O‐ring (9464K51, maximum temperature 400 °F or 204 °C; McMaster‐Carr) was located at the inlet end of the quartz tube. The chamber was first evacuated to 10^−2^ Torr base pressure, and then ultra‐high purity argon (Ar) (99.999%, O_2_ < 1 ppm, H_2_O < 1 ppm; Airgas USA, LLC) was introduced until atmospheric pressure. When the furnace temperature reached 1100 °C, silicon tetrachloride (SiCl_4_) (99%; Sigma‐Aldrich) vapor was introduced to the chamber by bubbling 80 sccm of 10% hydrogen (H_2_)‐balanced Ar gas (99.995%, O_2_ < 4 ppm, H_2_O < 3 ppm; Airgas USA, LLC) through a glass bubbler. After 0.5–3 min etching, ultrahigh‐purity Ar gas was streamed to cool down the chamber.

For the controlled fabrication of the Si nano/microstructure, a Si micro‐hole array pattern was prepared on SiO_2_ (300 nm)/Si wafer ((100)‐orientation, p‐type, resistivity 1–10 Ω cm; UniversityWafer, Inc.) using the photolithography followed by an inductively coupled plasma etching of SiO_2_. The diameter and spacing of the hole pattern were 5 μm and 10–100 μm, respectively. The as‐patterned SiO_2_/Si wafer was subsequently subjected to the CVE reaction. The etching process was the same as that of a bare Si wafer, except that the reaction time was controlled between 15 and 30 min, depending on the spacing and density of the Si micro‐window.

For the SERS measurement, the as‐prepared SiNWs/micro‐pit arrays were first treated with a diluted hydrogen fluoride (48%; Sigma‐Aldrich) solution with a concentration of 0.432% and subsequently dip‐coated by 0.04 m silver nitride (AgNO_3_) (≥ 99.9%; Sigma‐Aldrich) solution multiple times to uniformly decorate the nanowire arrays with AgNPs. After vacuum annealing at 500 °C, the AgNP‐coated SiNWs/micro‐pit arrays were used as the template for SERS detection.

### Materials Characterizations

The morphological and structural features of the SiNWs/inverted pyramid binary structures were characterized by a thermal‐field‐emission SEM (Supra 25 FE‐SEM; Zeiss). An aberration‐corrected transmission electron microscope (FEI Titan Themis 300) was used to determine the crystalline structures of the SiNWs formed inside etch pits. For HRTEM observations, the nanowires were dispersed in anhydrous ethanol (≤ 0.005% water; Sigma‐Aldrich) and dropped on a copper grid coated with lacey carbon. The reflectance of the nano/microstructured surface was measured using a UV–vis–NIR spectrophotometer (V770; Jasco) at room temperature in the wavelength range of 200–2500 nm. SERS measurement was performed using a Horiba Jobin Yvon HR800 Raman spectrometer. A 5 μL droplet of R6G solution (99%; Sigma‐Aldrich) with a concentration from 10^−11^ to 10^−6^ m was deposited onto the as‐prepared AgNP‐decorated substrate and let dry in a vacuum before Raman characterization. All Raman spectra and maps were recorded at the excitation wavelength of 532 nm using 50X and 100X microscope objective lenses with numerical aperture (NA) = 0.75 and 0.9, respectively. The hole size was 100 μm, and the acquisition time was set to 800 ms with 5 accumulations.

## Conflict of Interest

The invention described in this paper is the subject of a patent (US 9840774 B2) on December 12, 2017. The authors declare no further competing interests.

## Author Contributions

**Juyeon Seo**: conceptualization (equal); data curation (lead); formal analysis (lead); investigation (lead); methodology (lead); project administration (lead); validation (lead); visualization (lead); writing—original draft (lead); writing—review and editing (lead). **Peiyun Feng**: data curation (equal); formal analysis (equal); investigation (equal); methodology (equal); validation (equal); visualization (equal); writing—review and editing (supporting). **Jianlin Li**: data curation (supporting); investigation (equal); visualization (equal); writing—review and editing (supporting). **Sanghyun Hong**: conceptualization (equal); data curation (equal); investigation (equal); methodology (equal). **Sen Gao**: conceptualization (equal); data curation (equal); investigation (equal);project administration (equal); supervision (equal); writing—review and editing (equal). **Ji Young Byun**: validation (supporting). **Yung Joon Jung**: funding acquisition (lead); investigation (equal); project administration (equal); supervision (lead); writing—original draft (supporting); writing—review and editing (lead).

## Supporting information

Supplementary Material

## Data Availability

The data that support the findings of this study are available from the corresponding author upon reasonable request.
