# Supplementary material for: Scalable Fabrication of Highly Organized, Horizontally Aligned Sub‐5 nm Silicon Nanowires via Chemical Vapor Etching
Source: Small Sci. 2025 Feb 28;5(6):2400627. doi: 10.1002/smsc.202400627 (PMC12168600; doi:10.1002/smsc.202400627)
Supplement: Supplementary file 1 — Supplementary Material [file SMSC-5-2400627-s001.pdf]

Supporting Information

Scalable Fabrication of Highly Organized, Horizontally Aligned Sub-5 nm Silicon Nanowires via Chemical Vapor Etching

Juyeon Seo, Peiyun Feng, Jianlin Li, Sanghyun Hong, Sen Gao, Ji Young Byun, Yung Joon Jung**

Supplementary Note 1: Quantitative analysis of the size and density distribution of AgNPs on SiNW arrays.

The analysis of the size and density distribution of AgNPs was conducted on high-magnification SEM images using Photoshop and ImageJ software. First, the brightness and contrast of the SEM images taken at the magnification of 100 kX were adjusted by Photoshop software to enhance the visibility of AgNPs. Each image was then divided into 20 equal sections, each with an area of 5.85×10^2 μm^2. Sections exhibiting non-uniform AgNP distribution or inconsistent contrast were excluded from the statistical analysis. Following this, the contrast threshold of each section was adjusted using Image J software to highlight the AgNPs against the background. Based on this contrast threshold, the particles sized between 0.785 nm^2 (corresponding to a 1 nm diameter) and 706 nm^2 (corresponding to a 30 nm diameter) were automatically identified. This analysis did not impose restrictions on the circularity of the nanoparticles. For statistical calculations, the software measured area covered by each particle, assuming that AgNPs are perfect circles to estimate their diameter distributions. Lastly, the areal density of AgNPs was estimated as the number per square micrometer for the purpose of cross-sample comparison.

Supplementary Note 2: Determination of the number of probe molecules within the Raman active window.

The number of probe molecules present within the effective Raman laser scattering area (N_{SERS}) was determined using the following equation[1]:

$$N_{SERS} = \frac{C V N_A A_{Raman}}{A_{Substrate}}$$

where C represents the molar concentration of the probe molecules ($C = 10^{-11}$ M), V is the total volume of the probe solution applied to the SERS substrate ($V = 5$ μL), and N_A is Avogadro's constant. The Raman laser focusing area (A_{Raman}) was calculated based on the laser spot diameter, which was driven using the following equation[2]:

$$Laser\ spot\ diameter = 1.22 \times \frac{Laser\ wavelength}{Numeric\ aperture}$$

Raman spectra were recorded at the excitation wavelength of 532 nm using a 100X microscope objective lens with a numeric aperture of 0.90. Thus, the estimated laser spot diameter was 721 nm. The effective area of the substrate ($A_{Substrate}$) was determined by the area of the droplet spreading over the surface, which has a diameter of 3.4 mm.

Using these values, the N_{SERS} for our experimental setup was calculated as follows:

$$N_{SERS} = \frac{10^{-11} \times 5 \times 10^{-6} \times 6.022 \times 10^{23} \times \pi(0.5 \times 721 \times 10^{-9}m)^2}{\pi(1.7 \times 10^{-3}m)^2} = 1.35$$

The resultant value of N_{SERS} was 1.35, indicating that at the probe molecule concentration of 10^{-11} M, nearly a single molecule is present within the Raman active window. Therefore, the SERS signal obtained under these conditions can be interpreted as indicative of single-molecule detection.

Supplementary Note 3: Calculation of the enhancement factor.
The enhancement factor (EF) was determined as shown below:

$$EF = \frac{I_{SERS}}{I_{Bulk}} \times \frac{N_{Bulk}}{N_{SERS}}$$

where I_{SERS} and I_{Bulk} represent the enhanced and normal Raman signal intensity of R6G molecules at 611 cm^{-1}, respectively, while N_{SERS} and N_{Bulk} denote the number of probe molecules contributing to the enhanced and normal signal intensity, respectively.[1]

For the purpose of calculating EF for a single R6G molecule, N_{SERS} was set to 1.

The value of N_{Bulk} was determined using the following equation:

$$N_{Bulk} = \frac{\rho h N_A A_{Raman}}{M}$$

where h is the confocal depth of the laser beam, (h = 0.7 μm), and ρ and M represent the density and molecular weight of R6G, which are 1.26 g cm^{-3} and 479.02 g mol^{-1}, respectively. Thus, the resultant value of N_{Bulk} was 4.527×10^8.

Using these values, the enhancement factor (EF) was calculated as follows:

$$EF = \frac{I_{SERS}}{I_{Bulk}} \times \frac{N_{Bulk}}{N_{SERS}} = \frac{125.4}{1} \times \frac{4.527 \times 10^8}{1} = 5.677 \times 10^{10}$$

Thus, the resulting EF at 611 cm^{-1} was estimated to be 5.677×10^{10} at the concentration of 10^{-11} M.

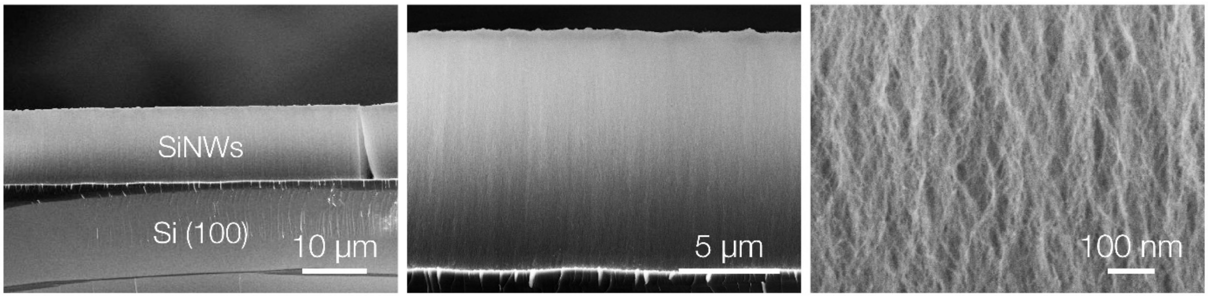

Figure S1. Low- and high-magnification SEM images of the vertically aligned SiNWs, directly formed on the silicon surface with high uniformity.

In our previous work, we demonstrated the synthesis of the <100>-oriented vertical SiNWs using the same Si (100) wafer.[3, 4] It was confirmed that the Si native oxide undergoes thermal decomposition during heating, followed by etching into a highly porous, high-density clustered structure by vapor etchants introduced at the target temperature range of 1000–1150 °C. Subsequently, a long-lasting continuous supply of etchants, maintained for over an hour at a lower H_2/Ar carrier gas flow of 20 sccm, facilitates homogenous etching of the Si surface through the porous oxide layer, leading to the uniform formation of the vertical nanowire structures **(Figure S1)**.

In contrast, the formation of horizontal SiNWs/micro-pit structure requires a higher etching kinetics from a non-uniform distribution of etchant vapors; thus, the reaction is completed over a much shorter time scale, typically a few minutes, at a higher carrier gas flow rate of 80 sccm. This variation in etchant supply results in an inhomogeneous distribution and localized concentration of HCl and $SiCl_4$ vapors in more thermally decomposed and weakened regions of the Si surface, which are more susceptible to localized attack. Consequently, these regions evolve into pit structures, which promote the formation of highly organized horizontal SiNW arrays **(Figure 2)**.

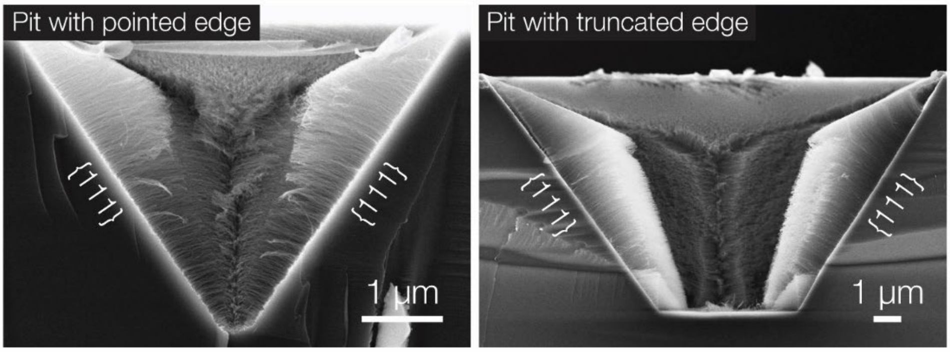

Figure S2. Cross-sectional SEM images of the horizontal SiNWs/micro-pits with pointed (left) and truncated (right) edges, bounded by Si {111} planes.

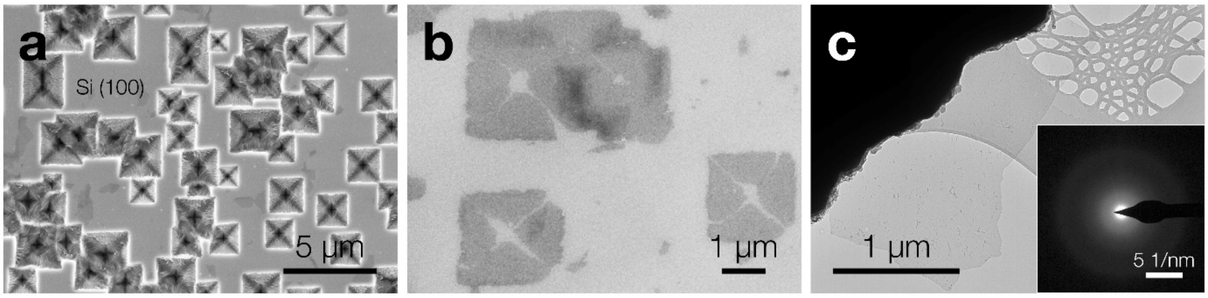

Figure S3. (a) Top-view SEM image of the SiNWs/micro-pits randomly distributed on the Si (100) surface after removing the top native oxide layer by a PDMS stamp. (b) SEM image of the cracked native oxide layer transferred onto SiO_2 (300 nm)/Si substrate for HRTEM characterization. The native oxide layer was first exfoliated from the SiNWs/micro-pits by the PDMS stamp, which was subsequently brought in contact with the target SiO_2/Si substrate. Then, the PDMS stamp was slowly peeled off, leaving the exfoliated native oxide layer. (c) Low-magnification HRTEM image of the native oxide layer covering the micro-pits. The inset is the SAED pattern of the native oxide layer, showing its amorphous characteristic.

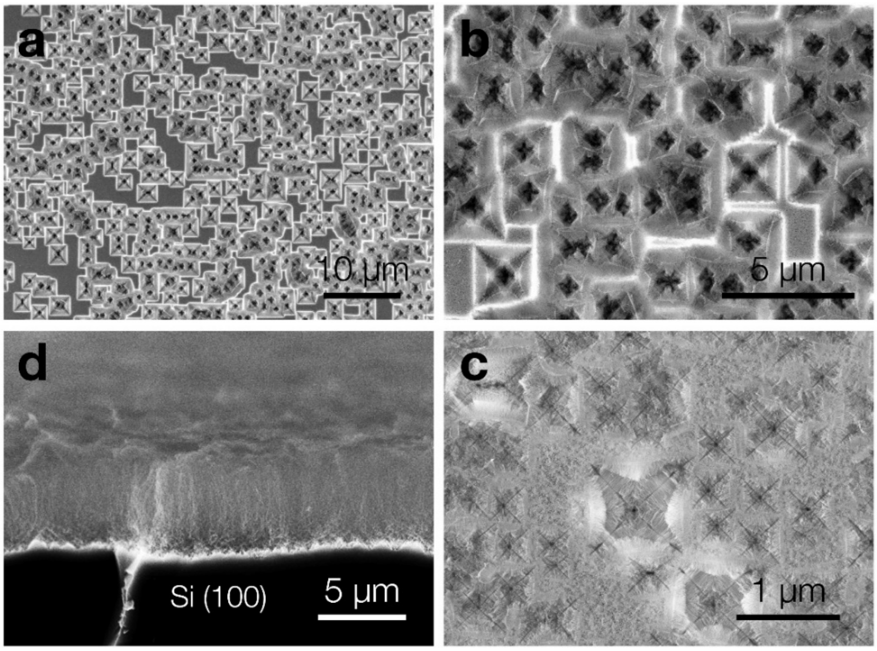

Figure S4. (a–c) Top-view SEM images showing the collapse of the pits over time. (d) Cross-sectional SEM image showing the vertical nanowires form after the collapse of the pits. The top rough surface is the trace of the morphology of the horizontal SiNWs/micro-pits.

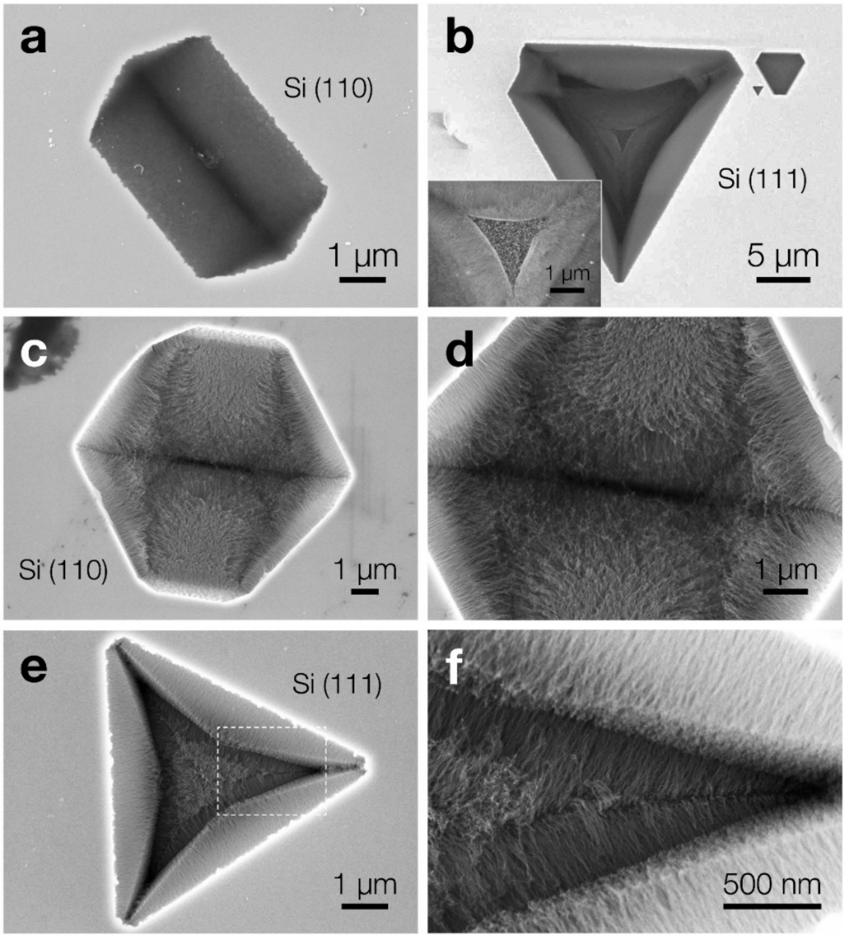

Figure S5. (a, b) Representative SEM images of the as-formed SiNWs/micro-pit covered by native oxide layer on (a) <110> and (b) <111>-oriented Si wafers. (c–f) Top-view SEM images of the SiNWs/micro-pit on the Si (c, d) {110} and (e, f) {111} surfaces after removing the native oxide layer with adhesive tape, showing the sub-5 nm nanowire arrays densely packed inside the micro-pits.

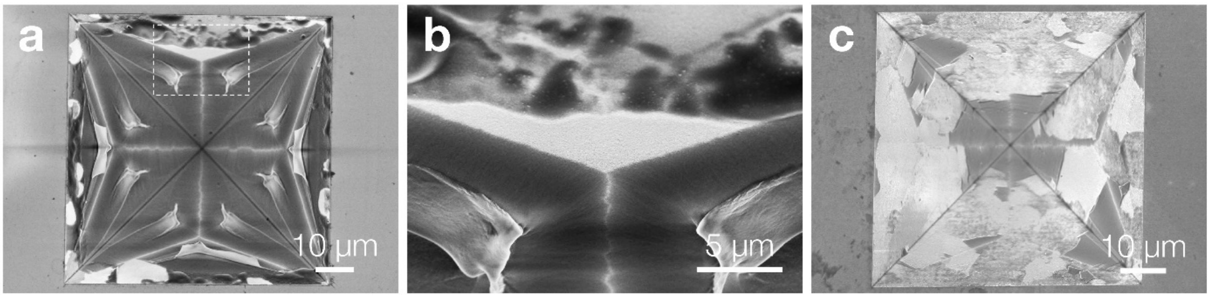

Figure S6. SEM image of the SiNWs/micro-pit structure after (a, b) partially and (c) completely removing the as-formed nanowires. It shows that the nanowires are organized in three different directions from the centroid of each triangular {111} facet.

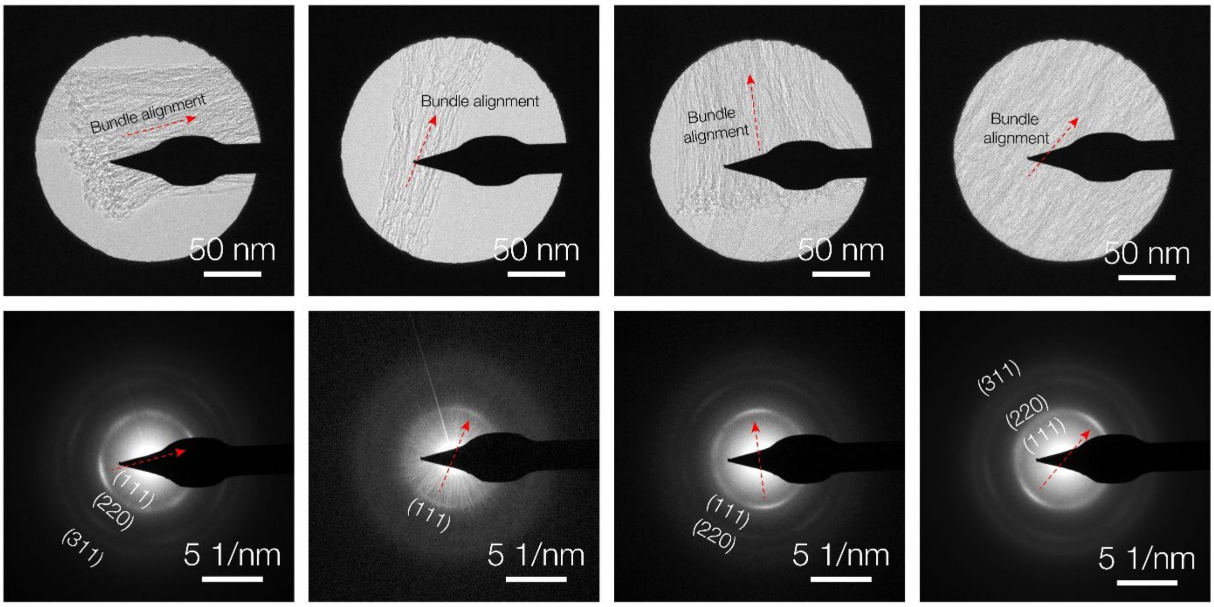

Figure S7. HRTEM images (top) and corresponding SAED patterns (bottom) of the SiNW bundles detached from the sidewalls of the micro-pits, confirming the <111> orientations of the SiNWs. The red arrows indicate the alignment of bundles.

During the CVE process, vapor etchants aggressively attack and remove surface Si atoms, resulting in rough sidewall morphologies of the SiNWs (i.e., sidewalls that are not perfectly straight) and introducing defects that isolate single-crystalline domains.[3] Furthermore, the ultra-narrow diameters of the nanowires make them highly susceptible to bending during TEM sample preparation processes, such as ultrasonic agitation and suspension of SiNWs. Consequently, while the nanowires within the bundles exhibit some alignment in one direction, this alignment is not perfect. The wide spots shown in **Figure 3c and Figure S7** arise from the overlap of diffraction spots from individual nanowires, which are slightly misaligned within the bundles.

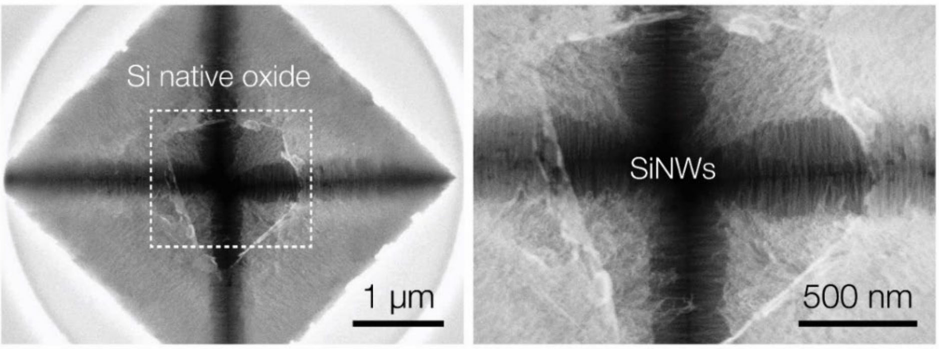

Figure S8. Enlarged SEM images of the individual etch pit created at Si micro-window, showing the damaged native oxide layer covering the as-generated etch pit. The horizontally aligned nanowires are also formed within the pit.

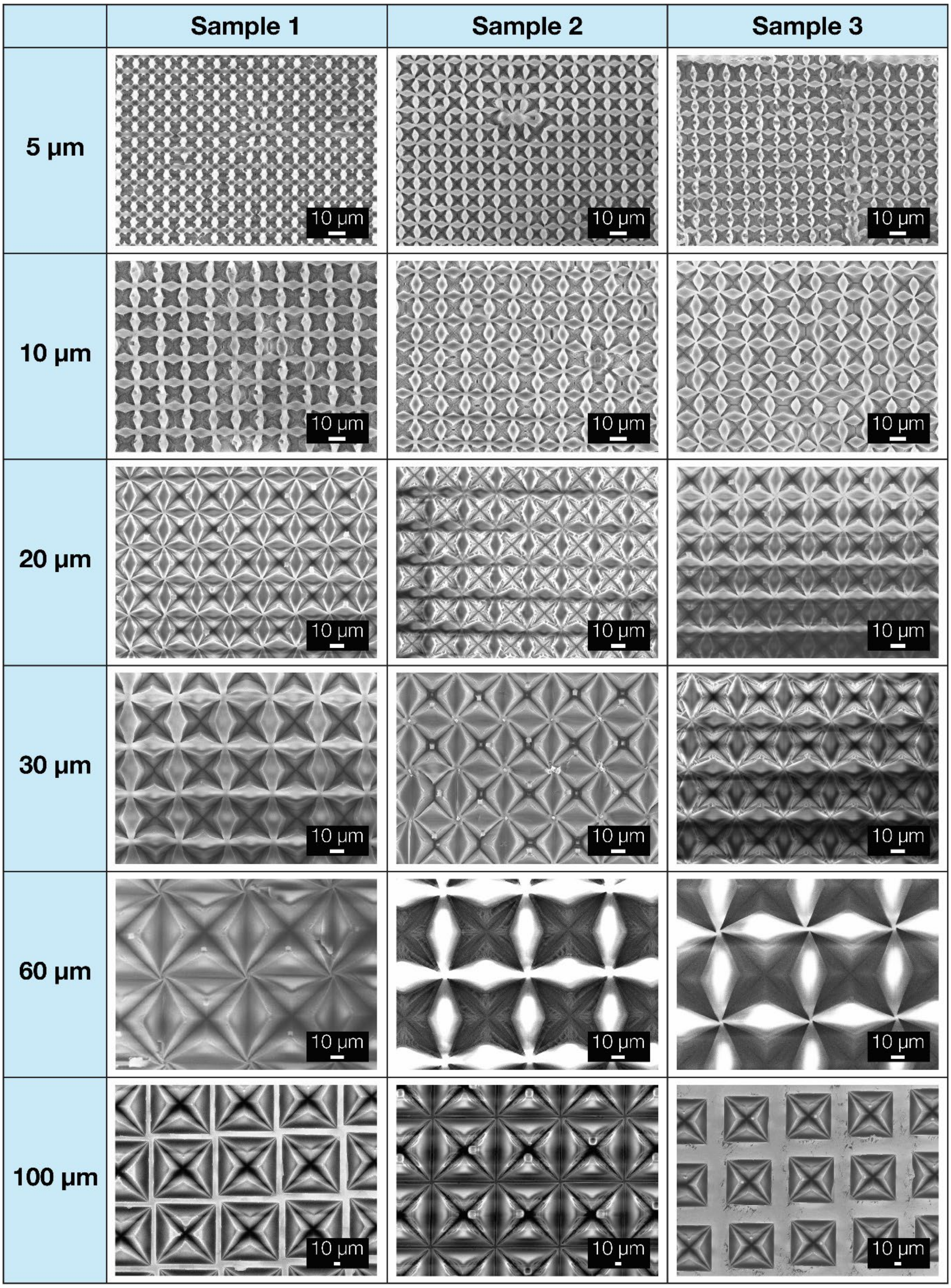

Figure S9. Top-view SEM images of three different Si hierarchical samples, demonstrating high uniformity and reproducibility of the template-based CVE process. The sizes, densities, and spacings of the SiNWs/micro-pit arrays were precisely controlled using micro-patterned SiO_2/Si templates with hole spacings ranging from 5 μm to 100 μm.

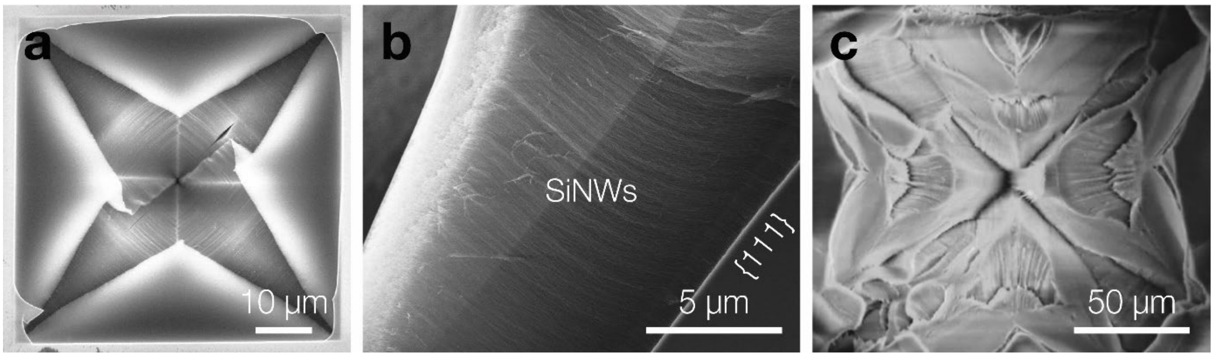

Figure S10. (a) Top and (b) cross-sectional SEM images of the SiNWs/micro-pit structure after a 30-minute CVE reaction. (c) Top-view SEM image of the SiNWs/micro-pit structure obtained after over 1-hour reaction. The results demonstrate that the SiNW arrays with high aspect ratios exceeding 10,000 can be obtained by optimizing the CVE reaction parameters.

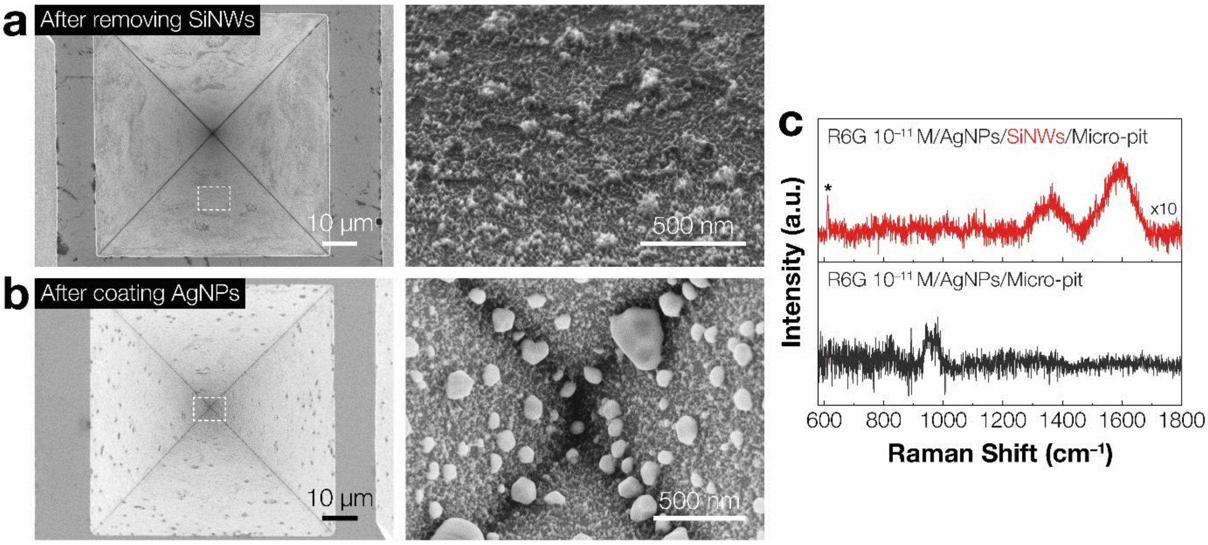

Figure S11. (a, b) Top-view SEM images of the micro-pit: (a) after removing the SiNWs and (b) after coating the AgNPs. The images on the right-hand side are the enlarged view of the region outlined by the white dashed lines. (c) Raman spectra of 10^{-11} M R6G on the AgNP-decorated micro-pits with (red) and without (gray) the SiNW arrays.

To assess the role of the sub-5 nm SiNW arrays, we performed additional experiments comparing the SERS performance of AgNP-decorated SiNWs/micro-pit structures with AgNP-decorated micro-pit without the SiNWs. The SiNWs were mechanically removed from the micro-pits using 3M Magic Tape, followed by bath sonication. As shown in **Figure S11a**, this process resulted in a roughened micro-pit surface with only the remnants of the short nanowires'

roots. Subsequently, we prepared the AgNP-decorated micro-pit following the same experimental procedures outlined in the Experimental Section of the main manuscript. **Figure S11b** demonstrates that the spatial distributions of the AgNPs differ significantly from those observed in the micro-pits with SiNWs. Specifically, without the SiNWs, the AgNPs display much larger diameters (61.91 ± 37.04 nm) and aggregate unevenly, leading to a lower areal density of 22.82 μm^{-2}. This result suggests that the formation of extremely small and highly dense AgNPs is primarily attributed to the sub-5 nm SiNW arrays rather than the rough surface of the micro-pits. The ultra-small diameter and high aspect ratio of the SiNWs facilitate the saturation of Ag ions at all available nucleation sites on the nanowires' surfaces while simultaneously restricting the growth of the AgNPs to dimensions on par with the nanowires themselves. Consequently, under the same SERS measurement conditions (as outlined in the Experimental Section), no Raman signal from R6G was observed from the AgNP-decorated micro-pit structures without the SiNWs (**Figure S11c**). On the other hand, two prominent R6G peaks at 611 cm^{-1} and 1360 cm^{-1} were clearly detectable in the AgNP-decorated SiNWs/micro-pits, even at an extremely low R6G concentration of 10^{-11} M. These findings indicate that the sub-5 nm SiNWs are crucial for enhancing SERS sensitivity. Their structural advantages facilitate denser and more uniform nucleation of the plasmonic AgNPs and promote strong near-field coupling between plasmonic nanostructures, further amplifying the SERS signal.

Table S1. Surface bond density and surface free energy of silicon crystal planes.[5, 6]

Surface orientation	Surface bond density [$\times 10^{15}$ cm^{-2}]	Surface energy [J cm^{-2}]
(100)	1.36	1.99
(110)	0.96	1.41
(111)	0.78	1.15

The higher surface bond density and surface free energy of the Si {100} planes make the surface atoms on these planes more reactive to the HCl and $SiCl_4$ vapor etchants, resulting in a faster etching rate (i.e., Si atom removal rate) along the <100> direction. Consequently, the etching process preferentially proceeds along the <100> directions until the more stable {111} planes with lower surface energy are exposed. This anisotropic behavior leads to the formation of square-shaped, inverted pyramidal structures bounded by {111} facets.

References

[1] S. Cong, Z. Wang, W. Gong, Z. Chen, W. Lu, J. R. Lombardi, Z. Zhao, *Nat. Commun.* **2019**, *10*, 678.
[2] M. Born, E. Wolf, A. B. Bhatia, *Principles of Optics: Electromagnetic Theory of Propagation, Interference and Diffraction of Light*, Cambridge University Press, Cambridge, UK 1999.
[3] S. Gao, S. Hong, S. Park, H. Y. Jung, W. Liang, Y. Lee, C. W. Ahn, J. Y. Byun, J. Seo, M. G. Hahm, H. Kim, K. Kim, Y. Yi, H. Wang, M. Upmanyu, S.-G. Lee, Y. Homma, H. Terrones, Y. J. Jung, *Nat. Commun.* **2022**, *13*, 3467.
[4] S. Gao, J. Seo, S. Hong, J. Li, P. Feng, J. Y. Byun, Y. J. Jung, *J. Mater. Chem. C* **2023**, *11*, 5102.
[5] P. J. Hesketh, C. Ju, S. Gowda, E. Zanoria, S. Danyluk, *J. Electrochem. Soc.* **1993**, *140*, 1080.
[6] C. Battaglia, G. Onida, K. Gaál-Nagy, P. Aebi, *Phys. Rev. B* **2009**, *80*, 214102.
